# Invasive Squamous Cell Carcinoma of the Larynx in a Dog

**DOI:** 10.1155/crve/5935429

**Published:** 2026-07-10

**Authors:** Edenilson Doná Frigerio, Samira das Mercês Saboia Bezerra, Luísa Husz Toledo, Bruna Rodrigues Ferreira Sengue, Maria Eduarda Roselli Silvério, Igor Luiz Salardani Senhorello, Alexandre Lima de Andrade, Daniela Bernadete Rozza, Gisele Fabrino Machado

**Affiliations:** ^1^ Department of Veterinary Clinics, Surgery and Reproduction, São Paulo State University (UNESP), School of Veterinary Medicine, Araçatuba, São Paulo, Brazil, unesp.br; ^2^ Hospital Veterinário Luiz Quintiliano de Oliveira, São Paulo State University (UNESP), School of Veterinary Medicine, Araçatuba, São Paulo, Brazil, unesp.br

**Keywords:** dog, electrochemotherapy, frozen section, laryngeal neoplasm, surgical margins

## Abstract

Squamous cell carcinoma (SCC) is a malignant epithelial neoplasm commonly affecting the skin and oral cavity of dogs, whereas laryngeal involvement is considered rare. Neoplasms arising in the larynx may cause severe upper airway obstruction and present diagnostic and therapeutic challenges due to the anatomical and functional constraints of this region. An 11‐year‐old neutered male mixed‐breed dog was evaluated for a one‐month history of progressive upper airway obstruction characterized by dyspnea, tachypnea, and dysphagia. Clinical examination revealed a solitary mass protruding into the laryngeal lumen in the arytenoid region. Surgical excision was performed, and intraoperative frozen section evaluation indicated compromised surgical margins. Definitive histopathology confirmed an invasive SCC. Adjunct electrochemotherapy was applied following identification of compromised surgical margins, and the dog showed favorable short‐term clinical recovery following surgery. No evidence of recurrence or metastasis was observed during a three‐month follow‐up period. This case highlights the clinical relevance of laryngeal SCC in dogs and emphasizes the importance of thorough clinical examination and early detection, as well as the integration of clinical evaluation, histopathology, and intraoperative margin assessment in the management of upper airway neoplasms. The combined use of surgical excision, frozen section analysis, and adjunct electrochemotherapy may be considered a potential therapeutic approach in anatomically constrained regions where complete tumor removal is limited.

## 1. Introduction

Squamous cell carcinoma (SCC) is a malignant epithelial neoplasm derived from keratinocytes and is characterized by locally infiltrative growth, variable degrees of squamous differentiation, and generally low to moderate metastatic potential in dogs [1]. It is one of the most frequently diagnosed malignant tumors in the canine species, primarily affecting the skin, oral cavity, and mucocutaneous regions, with biological behavior influenced by the anatomical site, degree of differentiation, and local extent of the lesion [1, 2].

Primary laryngeal neoplasms are considered rare in dogs, with most cases described in small case series or isolated reports [3, 4]. Available data indicate that these tumors predominantly affect adult to older dogs, although limited case numbers hinder the identification of consistent breed predispositions [4, 5].

Primary neoplasms affecting the larynx and other structures of the upper airway are considered uncommon in dogs, and the available literature is largely limited to isolated case reports, small case series, and retrospective reviews [6–10]. Among epithelial tumors described in these locations, SCC is one of the most frequently reported types [8–10]. Owing to the critical role of these structures in airflow conduction, masses in this region often result in progressive upper airway obstruction, including dyspnea, stridor, coughing, and exercise intolerance [7, 8, 11].

The diagnosis and therapeutic management of laryngeal neoplasms pose clinical and surgical challenges, mainly due to anatomical limitations that restrict the achievement of wide surgical margins without compromising essential functional structures [11]. Definitive diagnosis relies on histopathologic evaluation, whereas surgical margin assessment is clinically relevant in the management of SCC [1]. In this context, intraoperative frozen section analysis may serve as an adjunct tool in surgical decision‐making, allowing immediate identification of compromised margins and supporting the indication of adjuvant therapies when necessary [12].

Despite the clinical relevance of these lesions, there is a scarcity of detailed reports integrating clinical presentation, intraoperative findings, histopathologic evaluation, and therapeutic strategies for SCC involving the upper airway in dogs [8, 9]. Therefore, the present study is aimed at reporting a case of laryngeal SCC in a dog, emphasizing the clinical and histopathologic aspects and the role of intraoperative margin assessment in therapeutic planning.

## 2. Case Presentation

An 11‐year‐old, 6.2‐kg, neutered male mixed‐breed dog was referred to the Veterinary Hospital of São Paulo State University with an approximately 1‐month history of progressive upper airway respiratory signs. According to the owner, the dog exhibited episodes of reverse sneezing, dyspnea, and tachypnea, which were more pronounced during periods of excitement or stress, along with decreased exercise tolerance. Appetite was maintained; however, dysphagia and intermittent respiratory difficulty during feeding were observed.

On physical examination, the dog was tachypneic, alert, and responsive. Oral cavity inspection revealed a solitary mass protruding into the laryngeal cavity, located in the region of the arytenoid cartilage, which was difficult to access and precluded accurate assessment of its full extent (Figure 1A). On palpation, mild enlargement of the mandibular lymph nodes was noted. Complete blood count and serum biochemistry profile results were within reference ranges (Table 1). Thoracic radiographs were performed as part of the clinical evaluation due to respiratory signs and did not reveal radiographic abnormalities or evidence of pulmonary metastasis. Abdominal ultrasonography was also performed and showed no abnormalities. Mandibular lymph nodes were assessed by palpation and cytological examination, which did not reveal cytological abnormalities or evidence of metastatic involvement.

**Figure 1:**
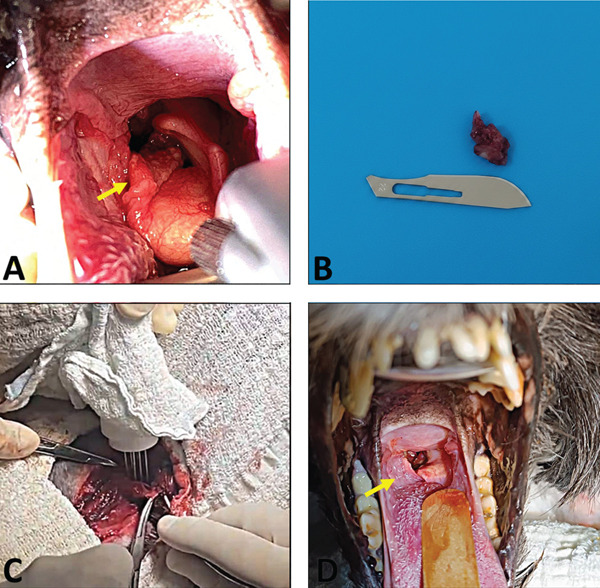
Clinical, intraoperative, and postoperative images of laryngeal squamous cell carcinoma in a dog. (A) Presence of the tumor in the laryngeal region (yellow arrow). (B) Surgically excised tumor, with a no. 22 scalpel blade used as a scale reference. (C) Application of electrochemotherapy to the surgical bed by laryngotomy using needle electrodes connected to an E‐PORE pulse generator (ANIMAL TECH, São Paulo, Brazil). (D) Postoperative appearance 10 days after the surgical procedure and electrochemotherapy, showing absence of visible neoplastic tissue and the macroscopic appearance of the larynx (yellow arrow).

**Table 1:** Hematologic and serum biochemical data obtained from an 11‐year‐old dog evaluated for progressive upper airway obstruction. Results are presented alongside corresponding laboratory reference intervals.

Variable	Result	Reference interval
Red blood cells (×10^6^ cells/*μ*L)	5.64	5.5–8.5
Hemoglobin (g/dL)	13.0	12.0–18.0
Hematocrit (%)	40	37–55
Mean corpuscular volume (fL)	70.92	60–77
Mean corpuscular hemoglobin concentration (g/dL)	32.5	28.1–35.8
Platelets (×10^3^ platelets/*μ*L)	484	175–500
White blood cells (×10^3^ cells/*μ*L)	12.0	6.0–17.0
Segmented neutrophils (×10^3^ cells/*μ*L)	10.56	3.3–11.5
Lymphocytes (×10^3^ cells/*μ*L)	1.06	1.0–4.8
Monocytes (×10^3^ cells/*μ*L)	0.26	0.15–1.35
Eosinophils (×10^3^ cells/*μ*L)	0.12	0.10–1.25
Basophils (×10^3^ cells/*μ*L)	0.00	0.00–rare
Alanine aminotransferase (IU/L)	58	21–102
Alkaline phosphatase (IU/L)	126	20–156
Total protein (serum) (g/dL)	6.3	5.4–7.1
Albumin (g/dL)	2.8	2.1–3.6
Globulin (g/dL)	3.5	2.7–4.4
Creatinine (mg/dL)	1.1	0.5–1.5
Urea (mg/dL)	35	21–60

Given the severity of the respiratory signs and the presence of the laryngeal mass, the dog underwent surgical intervention. Differential diagnoses for the laryngeal mass included inflammatory lesions, laryngeal polyps, and other primary neoplasms of the upper airway, such as lymphoma and rhabdomyoma. Preoperative evaluation included clinical and cardiopulmonary assessment. The dog was alert, with a heart rate of 124 beats/min, respiratory rate of 80 breaths/min with dyspnea, systolic arterial pressure of 140 mmHg, and body temperature of 37.9°C. Cardiopulmonary auscultation was unremarkable. Based on the clinical findings, the patient was classified as American Society of Anesthesiologists (ASA) physical Status III.

Anesthetic induction was performed using propofol (5 mg/kg), ketamine (1 mg/kg), and lidocaine (1 mg/kg). Oxygen supplementation and ventilation were maintained through a tracheal tube using pressure‐controlled ventilation, and anesthesia was maintained with isoflurane throughout the procedure.

Following anesthetic induction, the surgical procedure was performed with the patient in dorsal recumbency and the neck in cervical extension. After airway stabilization via temporary tracheostomy, a ventral midline cervical skin incision was made. The cervical fascia was incised, and the sternohyoid muscles were separated by blunt dissection along the median raphe, exposing the thyroid cartilage. To access the laryngeal lumen and permit direct visualization of the mass, a ventral laryngotomy was performed through a longitudinal midline incision of the thyroid cartilage.

The neoplasm, located in the arytenoid cartilage region, was excised to the mucosal level (debulking technique), with a 0.5‐cm margin from macroscopically healthy tissue. The resection was limited to the mucosal and adjacent soft tissue layers, preserving the structural integrity of the laryngeal wall. No partial or complete resection of the arytenoid cartilage was performed. Although the arytenoid cartilage appeared distorted by the mass, it was preserved during surgery and was not intentionally included in the excised specimen.

During the procedure, intraoperative frozen section biopsy was performed to evaluate the lateral and deep margins of the surgical specimen. Processing of each margin required approximately 10 min to obtain an intraoperative diagnosis. While the deep margin was being evaluated, hemostasis was performed, followed by a brief pause in the procedure. After confirmation of margin involvement, further surgical resection was not considered feasible due to anatomical constraints, and electrochemotherapy (ECT) was initiated. Evaluation of the lateral margins was performed subsequently. Frozen sections were prepared using an adapted portable microtome with application of water and tetrafluoroethane gas to the surgical fragment for freezing, and the sections were stained with Romanowski‐type stain for intraoperative evaluation. The intraoperative frozen section technique is demonstrated (Video S1). The excised tissue consisted of an exophytic, friable, white nodule with multifocal brown areas, measuring approximately 2.2 × 1.0 cm (Figure 1B).

Intraoperative histological examination of frozen sections of the excised laryngeal mass revealed proliferation of neoplastic epithelial cells, consisting of polygonal to rounded cells with moderately abundant, basophilic to slightly eosinophilic cytoplasm, arranged in solid nests and islets, delimited by loose to moderately fibrous stroma (Figure 2). The cell clusters had an expansive and infiltrative architecture, consistent with an invasive lesion. Focal concentric keratinization was observed, resembling keratin pearls, a feature highly suggestive of squamous differentiation.

**Figure 2:**
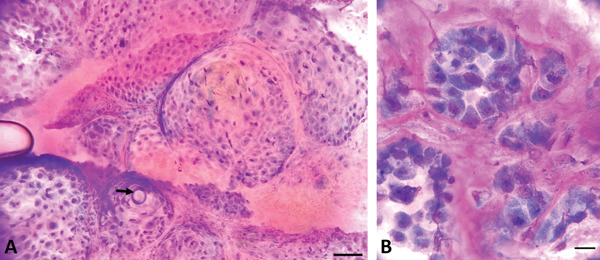
Photomicrographs of intraoperative frozen section of a laryngeal squamous cell carcinoma in a dog. (A) Frozen section showing nests of neoplastic epithelial cells with keratinization (arrow) (Romanowski‐type stain; bar = 50 *μ*m). (B) Higher magnification of the frozen section highlighting marked cellular atypia, including anisocytosis, anisokaryosis, pleomorphic nuclei, and increased nuclear‐to‐cytoplasmic ratio in neoplastic epithelial cells (Romanowski‐type stain; bar = 20 *μ*m).

Due to the identification of compromised surgical margins, adjunctive local ECT was instituted through the same surgical access used for tumor resection (Figure 1C). Adjunctive ECT was instituted in accordance with standard veterinary guidelines for the treatment of superficial neoplasms [13]. The protocol consisted of the systemic administration of intravenous bleomycin (Bonar, Biosintética, São Paulo, Brazil) at a dose of 15, 000 IU/m^2^ of body surface area. After an 8‐minute interval to ensure adequate drug diffusion into the tissues, electric pulses were initiated. An E‐PORE pulse generator (ANIMAL TECH, São Paulo, Brazil) was utilized, configured to deliver sequences of eight high‐voltage square pulses at 1300 V/cm, with a frequency of 5 kHz and a pulse duration of 100 *μ*s. The pulses were delivered through a needle electrode composed of two rows of three stainless steel needles (1.1 mm thick), with a 5 mm distance between contralateral needles and a 3 mm distance between ipsilateral needles. The electric field was applied to the entire tumor bed and surgical margins, ensuring a slight overlap between needle insertions to guarantee total area coverage and avoid “electrical shadow” zones that might lead to recurrence. The entire procedure was completed within the 40‐minute therapeutic window after chemotherapy administration to maintain maximum cytotoxic efficacy.

At the end of the ECT, the larynx was closed with simple interrupted sutures using a 2‐0 synthetic absorbable suture (Poliglactina 910, Ethicon J&J). The muscular layer and fascia were closed with a simple continuous pattern using a 2‐0 synthetic nonabsorbable suture (Mononylon, Ethicon J&J). Histopathologic evaluation identified the superficial aspect of the cartilage at the deep surgical margin, reflecting its close anatomical relationship to the lesion.

The surgical specimen was fixed in neutral buffered 10% formalin and routinely processed for histological examination. Histologically, there was a diffuse, poorly defined, expansive neoplastic proliferation infiltrating adjacent cartilage, composed of malignant epithelial cells, forming cell nests without basement membrane delimitation within a thin stroma (Figure 3). The cells showed squamous differentiation, as evidenced by polygonal cells with broad, eosinophilic cytoplasm, distinct borders, broad, round to oval nuclei with loose chromatin, and multiple nucleoli. There was marked cellular atypia, characterized by anisocytosis, anisokaryosis, and a high nucleus–cytoplasm ratio, nuclear pleomorphism, along with karyomegaly, binucleation, and numerous atypical mitotic figures (55 mitotic figures/10 high‐power fields [400x]).

**Figure 3:**
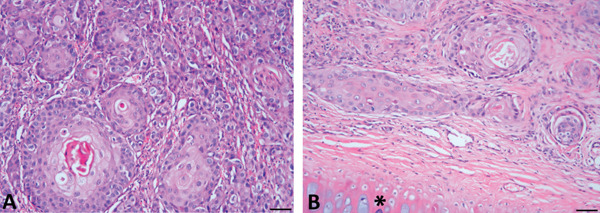
Photomicrographs of the paraffin histopathological examination of squamous cell carcinoma of the larynx in a dog. (A) Histological section showing an infiltrative proliferation of malignant epithelial cells arranged in nests and islands, with squamous differentiation and keratin pearl formation (H&E stain; bar = 50 *μ*m). (B) Neoplastic epithelial cell nests located adjacent to cartilaginous tissue (∗) (H&E stain; bar = 50 *μ*m).

The temporary tracheostomy was reversed 10 days after surgery with no recurrence of dyspnea or other respiratory signs during the immediate postoperative period. At that time, the oral cavity was reexamined, and no residual tumor tissue, marked necrosis, or suture dehiscence was observed, indicating a favorable local response and the absence of significant adverse effects (Figure 1D). The dog was monitored for 3 months following surgery through periodic clinical examinations and imaging studies, during which no evidence of local recurrence or metastatic disease was observed. After this period, the patient was lost to follow‐up. Although short‐term monitoring did not reveal evidence of recurrence or metastatic progression, the absence of long‐term follow‐up represents a limitation of this report and precludes definitive conclusions regarding treatment efficacy.

## 3. Discussion

The clinical presentation observed in this case, characterized by progressive dyspnea, tachypnea, reverse sneezing, exercise intolerance, and dysphagia, is consistent with previous reports of laryngeal neoplasms in dogs and reflects mechanical obstruction of the upper airway resulting from lesion location [7, 8, 11]. The presence of chronic respiratory signs in an elderly dog reinforces the need to include laryngeal neoplasia in the differential diagnosis, despite the low reported frequency of these lesions.

In this case, basic oncological staging was performed, including thoracic radiographs, abdominal ultrasonography, and cytological evaluation of mandibular lymph nodes, with no evidence of metastatic disease. However, the absence of advanced imaging techniques, such as computed tomography, may represent a limitation in fully assessing the extent of the disease.

The histopathologic findings were consistent with invasive SCC, characterized by infiltrative proliferation of malignant epithelial cells with squamous differentiation, including keratin pearl formation, cellular pleomorphism, and increased mitotic activity [1, 2]. These findings allowed confirmation of the morphologic diagnosis and exclusion of other epithelial or mesenchymal neoplasms described in the canine larynx.

Surgical management of laryngeal neoplasms is particularly challenging due to anatomical constraints that limit the achievement of wide surgical margins without significant functional compromise [11]. In this case, the identification of compromised surgical margins through intraoperative frozen section analysis directly influenced clinical decision‐making, supporting the indication of adjuvant ECT. Given the anatomical constraints of the laryngeal region, additional surgical resection to achieve wider margins was not considered feasible without significant functional compromise, and adjuvant therapy was therefore selected to improve local tumor control.

ECT has been increasingly used as an adjuvant therapeutic modality for SCC in veterinary medicine, with favorable response rates reported in previous studies [13, 14]. Although laryngeal SCC is considered rare in dogs, and therapeutic protocols for this site are often extrapolated from studies involving cutaneous or oral SCC, the technique′s physical mechanism of action supports its potential applicability in the laryngeal region. [11, 13, 15].

In the present case, the anatomical and functional constraints of the larynx precluded the achievement of wide surgical margins. In this scenario, adjunct ECT was instituted specifically for the treatment of compromised surgical margins identified during intraoperative frozen section analysis. This approach is based on the selective toxicity of bleomycin for rapidly dividing neoplastic cells, although healthy, quiescent tissues at the margins remain viable even after electropermeabilization [13]. Such selectivity facilitates local disease control while preserving the structural integrity and essential functions of the larynx, which is fundamental for maintaining the patient′s quality of life [16].

To the authors′ knowledge, this is the first case to describe the combination of surgery and ECT for the management of laryngeal carcinoma in a dog. The clinical outcome demonstrates that the technique was safe for this sensitive anatomical site, as no postoperative complications—such as necrosis or suture dehiscence—were observed [15, 16]. The lack of recurrence during the follow‐up period supports the evidence that this synergistic approach provides robust local control in restricted regions where surgical radicality alone is unfeasible [17].

Although most of the evidence regarding frozen section analysis originates from human oncology, its principles have been progressively applied in veterinary medicine, particularly for locally invasive tumors [12]. Frozen section evaluation allows rapid intraoperative assessment of tumor margins and lesion characterization; however, it should be interpreted cautiously because technical limitations and sampling errors may occur, and definitive diagnosis still relies on conventional histopathology [18]. In the present case, integration of surgical resection, intraoperative margin assessment, and adjuvant therapy resulted in immediate clinical control of airway obstruction, with no recurrence of respiratory signs during the early postoperative period.

Although short‐term follow‐up (3 months) did not reveal evidence of recurrence or metastatic progression, the absence of long‐term follow‐up represents a limitation of this report and prevents definitive conclusions regarding disease‐free interval, overall survival, and treatment efficacy. These findings should therefore be interpreted with caution, particularly considering the inability to determine the individual contribution of each therapeutic modality.

This case report describes a rare laryngeal SCC in a dog and reinforces the importance of clinicopathologic integration for diagnosis and therapeutic decision‐making in upper airway tumors. The combined use of intraoperative margin assessment and adjuvant therapy is described in this case and may provide useful information for the management of similar lesions in anatomically challenging locations; however, these findings should be interpreted as preliminary observations.

## Funding

This study was financed by the Coordenação de Aperfeiçoamento de Pessoal de Nível Superior, 10.13039/501100002322.

## Ethics Statement

This report describes a clinical case managed according to institutional standards of veterinary care. Written informed consent was obtained from the owner for diagnostic procedures, treatment, and publication of this case.

## Conflicts of Interest

The authors declare no conflicts of interest.

## Supporting information


**Supporting Information** Additional supporting information can be found online in the Supporting Information section. Video S1: Intraoperative frozen section technique performed during surgical excision of a laryngeal squamous cell carcinoma in a dog. The video demonstrates rapid tissue freezing and preparation of sections for intraoperative margin assessment.

## Data Availability

Data sharing is not applicable to this article as no datasets were generated or analyzed during the current study.
